# HR9: An Important Cell Penetrating Peptide for Delivery of HCV NS3 DNA into HEK-293T Cells

**Published:** 2020

**Authors:** Sina Alizadeh, Shiva Irani, Azam Bolhassani, Seyed Mehdi Sadat

**Affiliations:** 1. Department of Biology, Faculty of Basic Sciences, Science and Research Branch, Islamic Azad University, Tehran, Iran; 2. Department of Hepatitis and AIDS, Pasteur Institute of Iran, Tehran, Iran

**Keywords:** Cell penetrating peptide, DNA, Gene delivery system, Hepatitis C virus, Vaccines

## Abstract

**Background:**

The delivery of exogenous genes into cells for functional expression is required for development of DNA vaccine and gene therapy in medicine and pharmacology. Cell Penetrating Peptides (CPPs) were considered to mediate gene and drug delivery into living cells. In this study, an attempt was made to evaluate the efficiency of an arginine-rich CPP, HR9, in HCV NS3 gene delivery compared to TurboFect cationic polymer and supercharged +36 GFP into HEK-293T cells.

**Methods:**

The recombinant pEGFP-NS3 was constructed and their accuracy was confirmed by digestion and sequencing. Then, the recombinant plasmid was transfected into HEK-293T cells by TurboFect, +36 GFP and HR9 gene delivery systems. The expression of NS3 protein was assessed by fluorescent microscopy, flow cytometry and western blotting.

**Results:**

Our data indicated that HR9 peptide was able to form stable complexes with plasmid DNA and increased its delivery into HEK-293T cells in a non-covalent manner. Furthermore, treatment of cells with HR9 and HR9/DNA complexes resulted in a viability of 90–95% indicating this CPP was not cytotoxic. The analysis of zeta potential and size showed the importance of interactions between positively-charged HR9/pEGFP-NS3 complexes and negatively-charged plasma membranes.

**Conclusion:**

The non-toxic HR9 CPP can be considered an effective carrier for delivering plasmid DNA harboring *Hepatitis C virus (HCV)* gene in therapeutic vaccine design.

## Introduction

*Hepatitis C virus (HCV)* has chronically infected an estimated 200 million people worldwide indicating one of the greatest public health threats of this century 1. The HCV proteome contains 3,011 amino acids, and is divided into three structural proteins (*i.e*., core, E1 and E2) and seven Non-Structural (NS) proteins (*i.e*., p7, NS2, NS3, NS4A, NS4B, NS5A and NS5B) 2,3. Some reports indicated the importance of a T cell-based vaccine against HCV infection due to strong association of broadly directed immune responses involving both CD4+ and CD8+ T cells with viral clearance 2,3. Among viral genes, the NS3 protein is highly conserved among different strains, carries multiple CD4+ and CD8+ T cell epitopes, and is the major target of T cell-mediated immunity which mediates HCV viral control in natural infection 4–6. The studies showed that the addition of adjuvants and/or delivery systems to the HCV proteins as an antigen enhanced serum antibody and T-cell pro-liferative responses as well as IFN-γ responses by CD4+ T cells in vaccine development^7^.

Among delivery systems, Cell Penetrating Peptides (CPPs) have attracted a special interest. They are positively-charged short peptide sequences, rich in lysine or arginine 8. The cell-penetrating peptides have the ability of permeating plasma membranes and carrying cargoes to enter cells 9. CPPs successfully delivered therapeutic peptides and proteins to target cells in treatment of human diseases such as cancer, diabetes, asthma, ischemia, *etc*. Most of these applications use CPPs (*e.g*., HIV-1 Tat, penetratin, polyarginine and HSV-1 VP22) covalently linked to peptides or as fusion proteins 10. As known, genetic vaccines especially DNA vaccines could induce both humoral and cellular immune responses in animals and human 11. Recently, the peptide-mediated DNA delivery system has become a novel vehicle for gene transfer in a non-covalent approach. For example, three different arginine-rich CPPs such as synthetic nona-arginine (SR9), histidine-rich nona-arginine (HR9) and Pas nona-arginine (PR9) showed the ability to transfer DNA into plant cells. These studies showed that CPPs alone or with their cargoes had no cytotoxicity at their doses of interest 9. Recently, superpositively charged Green Fluorescent Proteins (GFPs), including a variant with a theoretical net charge of +36 (+36 GFP) have been introduced to penetrate into various eukaryotic cell lines. These studies showed that +36 GFP is resistant to proteolysis, is stable in the presence of serum, and extends the serum half-life of siRNA and plasmid DNA. In fact, +36 GFP could mediate DNA transfection, enabling plasmid-based gene expression 12.

Up to now, HR9 peptide was used to deliver the reporter genes (*e.g*., wild type GFP), but its efficiency was not studied to deliver viral genes into the cells. In addition, the efficacy of a cell penetrating peptide (*e.g.*, HR9) and a cell penetrating protein (*e.g.*, +36 GFP) was not compared to deliver viral genes. In this study, our major goal was to determine the ability of an arginine-rich CPP, HR9, to deliver plasmid DNAs (pEGF-P-C3 and pEGFP-NS3) into a mammalian cell line (HEK-293T cells) and confirm the CPP-mediated gene delivery at the protein level for the first time. In this line, a commercial transfection reagent (*e.g.*, TurboFect), and a cell penetrating protein (*e.g.*, supercharged +36 GFP) 13 were used to compare the potency of NS3 DNA delivery into the cells versus HR9 CPP.

## Materials and Methods

### Peptide and plasmid preparation

A histidine-modified arginine-rich CPP (HR9, CH-HHHHRRRRRRRRRHHHHHC, MW: 3001.7 *Da*) was synthesized and purified by BioMatik Co. (Canada). To prepare the pEGFP-NS3, the eukaryotic vector (pc-DNA3.1) harboring the immunogenic and conserved region of HCV *NS3* gene (1095–1379 aa, previously provided in our laboratory) was digested by XhoI/ HindIII (Thermo scientific Fast digest) and subcloned into pEGFP-C3 expression vector. Purification of pEGFP-NS3 and pEGFP-C3 (as a positive control) was performed by ion exchange chromatography with an EndoFree Plasmid Mega Kit (Qiagen) according to the manufacturer’s instructions. The recombinant protein of +36 GFP was previously provided by our group^13^.

### Preparation and characterization of HR9 peptide/NS3 DNA nanoparticles

For preparation of peptide/DNA complexes, the peptide solution was added dropwise to 1 *μg* of plasmid DNA at different molar ratios of basic amino acid residues in the HR9 peptide to DNA phosphates (N/P ratio) to a final volume of 50 *μL* and incubated at room temperature for 30 *min*. One *μg* of the HR9 was estimated to have about 6.3 *nM* nitrogens and 1 *μg* of DNA was shown to include about 3 *nM* phosphates. Thus, the molar Nitrogen/Phosphate (N/P) ratios were determined 14, 15. The used ratios were 0, 1, 2, 5 and 10. The condensation between HR9 peptide and DNA was assessed by gel retardation assay. The gel retardation assay was performed for the pEGFP-C3/ HR9 complexes at the same ratios. To assess the stability of HR9/NS3 DNA complexes against DNA nucleases, DNase I was added to the complexes at different N/P ratios of 0, 2, 5 and 10 with a final concentration of 1.37 *U* enzyme/ 1 *μg* DNA and the mixtures were incubated at 37*°C* for 1 hr followed by the addition of stop solution (200 *mM* sodium chloride, 20 *mM* EDTA and 1% SDS) 16. Moreover, for evaluation of the serum stability, the nanoparticles at the N/P ratios of 0, 2 and 5 were exposed to 10% serum and incubated for 5 *hr* at 37*°C*. Then, DNA plasmids were released from protein by adding 10% SDS solution for 2 *hr* and analyzed with electrophoresis on agarose gel 1% 17. On the other hand, the size and zeta-potential of pEGFP-NS3 (or NS3 DNA) and the peptide/DNA complex at N/P ratio of 5:1 were measured by a Zetasizer Nano ZS instrument (Malvern Instruments, UK) at 25*°C*. Finally, the HR9/NS3DNA nanoparticles were prepared at N/P ratio of 5:1, and the size and morphology of nanoparticles were analyzed with a Scanning Electron Microscope (SEM) (KYKY-EM3200 model, China).

As a positive control, the supercharge +36 GFP solution was added to 1 *μg* of plasmid DNA (pEGFP-NS3) at different N/P ratios of 1:1, 2:1, 5:1, 10:1 and 20:1, and incubated for 15 min at room temperature 13. The condensation between +36 GFP and NS3 DNA was assessed by gel retardation assay. The +36 GFP/ NS3 DNA nanoparticles were prepared at N/P ratio of 10:1, and their size and morphology were analyzed with a SEM.

### HR9-mediated DNA delivery into HEK-293T cells

Human Embryonic Kidney cells (HEK-293T, Pasteur Institute of Iran) were grown in complete RPMI (Gibco) supplemented with 5% heat-inactivated Fetal Bovine Serum (FBS) (hi-FBS, Gibco) at 37*°C* in the presence of 5% CO_2_ atmosphere. The HEK-293T cell line was placed at a density of 4×104 cells/well in a 24-well plate (Greiner, Germany) in complete RPMI-1640 supplemented with 5% hi-FBS. The HR9/ pEGFP-NS3 nanoparticles at the N/P ratios of 2:1 and 5:1 were prepared and incubated for 30 *min* at room temperature in a total volume of 100 *μL*. Then, HR9/pEGFP-NS3 nanoparticles were added to the cells in serum-free medium. The medium was replaced after 1 *hr* incubation at 37*°C* with complete RPMI 5% hi-FBS. The pEGFP-NS3/TurboFect and pEGFP-C3/TurboFect complexes (Fermentas) were used as a positive control according to the manufacturer’s instructions. Briefly, for DNA transfection with TurboFect (cationic polymer, Fermentas), the recombinant pEGFP-NS3 and pEGFP-C3 vectors (∼1 *μg*) were pre-incubated with 4 *μL* of reagent in a final volume of 25 *μL* and incubated at room temperature for 20 *min* to form the DNA/TurboFect complexes. The complexes were then added to the cells in each well containing serum-free medium. The medium was replaced after 6 *hr* incubation at 37*°C* with complete RPMI 5% hi-FBS. On the other hand, +36 GFP/pEGFP-NS3 nanoparticles at N/P ratio of 10:1 were gently added to the cells in serum-free medium. The medium was replaced after 4 *hr* incubation at 37*°C* with complete RPMI 5% hi-FBS as previously reported 13. The transfection efficiency was monitored by fluorescence microscopy (EnvertFluorescent Ceti, Korea) and quantified by a FACSCalibur flow cytometer (Partec, Germany) 48 *hr* post-transfection.

For identification of NS3-GFP protein expression into the cells, western blotting was performed for the cells transfected with the HR9/pEGFP-NS3 (N/P=5:1) and +36 GFP/pEGFP-NS3 (N/P=10:1) complexes. The anti-GFP polyclonal antibody (Abcam; 1:10000 v/v) was used to confirm NS3 protein expression under standard procedures. The immunoreactive protein bands were visualized using peroxidase substrate named 3, 3’-diaminobenzidine (DAB, Sigma).

### Cytotoxicity assays

The MTT proliferation assay (Sigma) was utilized to evaluate the cytotoxicity of the naked DNA (pEGFP-NS3), the HR9/pEGFP-NS3 nanoparticles at different ratios of 1:1, 2:1, 5:1 and 10:1, and various molar concentrations of HR9 peptide (1, 2, 5 and 10) as well as 70% ethanol as a positive control in the non-malignant HEK-293T cell line. MTT assay was done in triplicate according to the manufacturer’s instructions.

### Statistical analysis

Student’s t-test was performed to analyze the percentage of nanoparticles transfection using flow cytometry and also cytotoxicity assay. Results were expressed as mean±standard deviation. The value of p< 0.05 was considered statistically significant. Similar results were obtained in two independent experiments.

## Results

### Characteristics of HR9 CPP/NS3 DNA complexes

**Figure 1.**
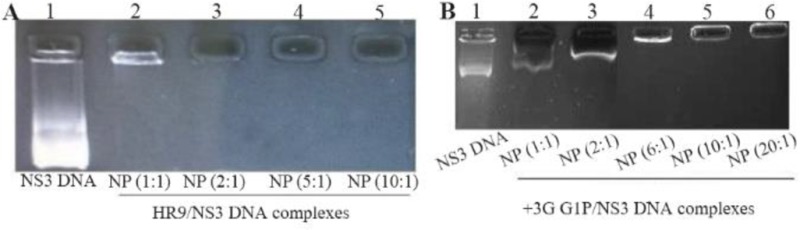
A) Representative gel retardation assay of HR9 peptide complexed with pEGFP-NS3 at different N/P ratios: Lane 1: naked plasmid DNA as a control (pEGFP-NS3), Lane 2: N/P = 1:1, Lane 3: N/P=2:1, Lane 4: N/P = 5:1, and Lane 5: N/P = 10:1; B) Gel retardation assay of +36 GFP complexed with pEGFP-NS3 at various N/P ratios: Lane 1: pEGFP-NS3, Lane 2: N/P = 1:1, Lane 3: N/P = 2:1, Lane 4: N/P=5:1, Lane 5: N/P = 10:1 and Lane 6: N/P = 20:1. The mixtures were analyzed by electrophoresis on a 1% agarose gel. The DNAs complexed with HR9 peptide and +36 GFP that were not able to migrate into the gels were observed at an N/P ratio of 2:1 and 5:1, respectively.

**Figure 2.**
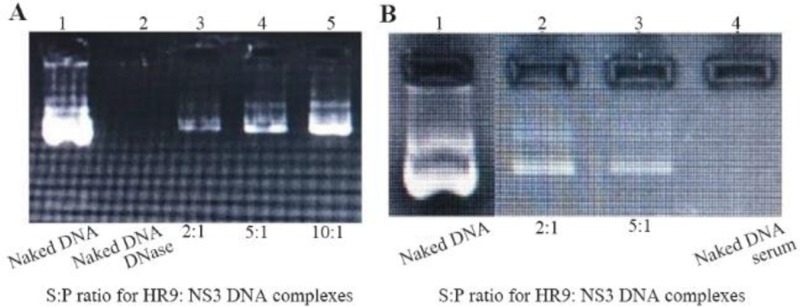
(A) Stability analysis of HR9-based nanoparticles against DNase I; Lane 1: naked plasmid DNA without DNase, Lane 2: naked plasmid DNA with DNase, Lane 3: N/P=2:1, Lane 4: N/P=5:1, Lane 5: N/P=10:1; (B) Stability analysis of HR9-based nanoparticles against serum; Lane 1: naked plasmid DNA, Lane 2: N/P=2:1, Lane 3: N/P = 5:1, Lane 4: naked plasmid DNA with serum.

**Figure 3.**
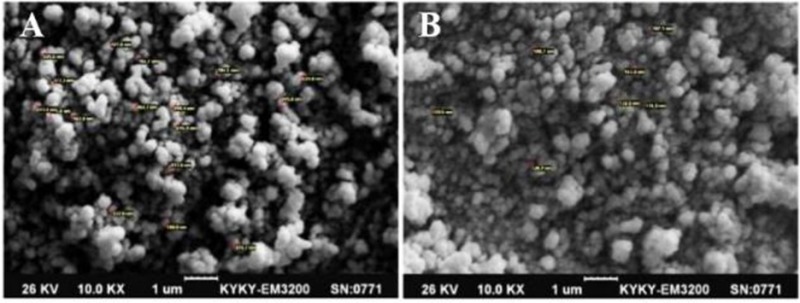
The SEM micrograph of the spherical +36 GFP/NS3 DNA nanoparticles formed at N/P= 10:1 (A), and HR9/NS3 DNA nanoparticles formed at N/P= 5:1 (B) with 10,000× magnification: A size of ∼200–250 *nm* was observed for +36 GFP/NS3 DNA nanoparticles and ∼100–150 *nm* for HR9/ NS3 DNA nanoparticles at 25°*C.*

At first, the NS3 DNA construct (*i.e*., pEGFP-NS3) was generated and confirmed. The recombinant pEGF-P-NS3 digested by XhoI and HindIII restriction enzymes showed a clear band of ∼861 *bp* related to the truncated *NS3* gene. The recombinant endotoxin-free plasmid had a concentration range between 1.7 and 2 *mg/ml*. To determine the interaction between HR9 and NS3 DNA, the recombinant pEGFP-NS3 was mixed with the increasing amounts of HR9 CPP to form various N/P ratios. It was found that the relative mobility of DNA was decreased when the ratio of HR9/DNA was increased. As shown in figure 1A, the DNA molecule did not migrate into the agarose gel at an N/P ratio of 2:1, indicating the formation of the HR9/pEGFP-NS3 complexes. The pEGFP-C3/HR9 complexes were also formed at N/P ratio of 1:1 (data not shown). Furthermore, the negatively charged pEGFP-NS3 plasmid interacted with the cationic+36 GFP for generation of nanoparticles. As shown in figure 1B, the DNA molecule did not migrate into the agarose gel at N/P ratio of 5:1, indicating the formation of the +36 GFP/pEGFP-NS3 complexes. For stability assay, after DNase I treatment, the naked NS3 DNA was quickly degraded, while the HR9/NS3DNA complexes protected the DNA from DNase I degradation at the N/P ratios more than 2:1 (Figure 2A). For serum protection assay, the N/P ratios of 2:1 and 5:1 were selected based on the analysis of DNase I stability and gel retardation. Agarose gel electrophoresis showed that unprotected plasmid DNA was degraded in the presence of serum after 5 *hr* incubation with hi-FBS. In contrast, recovered DNA from nanoparticles remained intact (Figure 2B). To analyze the size of complexed particles, the NS3 DNA and HR9 CPP/NS3 DNA complexes at N/P ratio (5:1) were measured using a Zetasizer. The NS3 DNA was approximately 396 *nm* (Z. Average: 396 *nm*) in diameter. Addition of HR9 CPPs to NS3 DNA increased the diameters of HR9/NS3 DNA complexes in water to about 853 *nm* (Z. Average: 853 *nm*). Their surface charges were also analyzed. NS3 DNA exhibited negative charges (−16.8 *mV*), while HR9/NS3 DNA complexes displayed positive charges (+8.82 *mV*). This suggests that electropositive charges of HR9 CPP/NS3 DNA complexes can be considered as an important factor for transport across the negatively -charged cytoplasmic membrane of HEK-293T cells. SEM analysis of nanoparticles showed a spherical shape with a size of ∼100–150 *nm* for the HR9/NS3 DNA nanoparticles (N/P: 5:1) and a size of ∼200–250 *nm* for +36 GFP/ NS3 DNA nanoparticles at 25*°C* (Figure 3).

**Figure 4.**
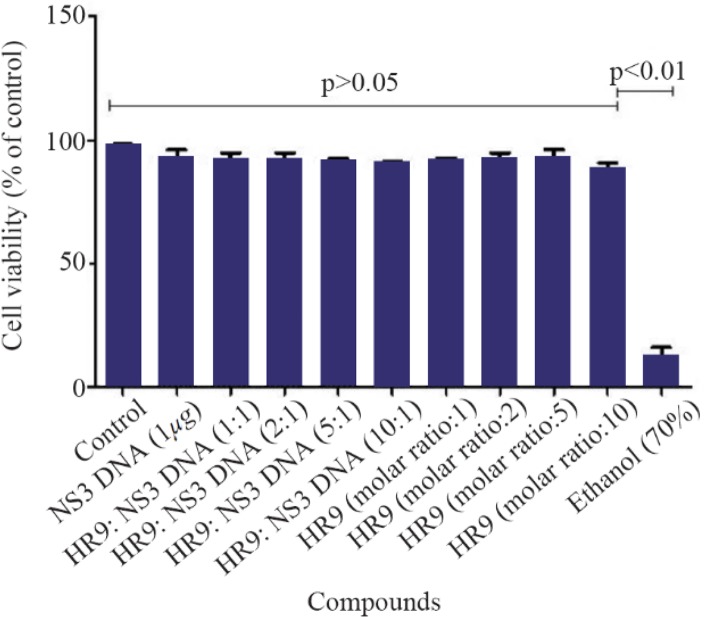
Cell viability of the HR9 and HR9 /NS3 DNA treatment. HEK-293T cells were treated with 1 μg of the naked DNA, HR9 CPP/DNA complexes at different N/P ratios of 1:1, 2:1, 5:1 and 10:1, HR9 CPP at molar ratios of 1, 2, 5 and 10 as well as 70% ethanol as a positive control. The MTT assay was used to evaluate cytotoxicity. Data were presented as mean±standard deviations from two independent experiments.

According to MTT results, the HR9 peptides, and HR9/pEGFP-NS3 complexes at N/P ratios of 1:1, 2:1, 5:1 and 10:1 did not induce any considerable cytotoxic effect compared to untreated cells (control, Figure 4) over a period of 48 *hr*. Treatment of the cells with the HR9 and HR9/DNA complexes showed cell viability of 90–95% (p>0.05). However, the cells treated with 70% ethanol indicated significantly strong decrease in viability (∼12%) compared to other groups (p< 0.01).

### Delivery of NS3 DNA by HR9 peptide into HEK-293T cells

**Figure 5.**
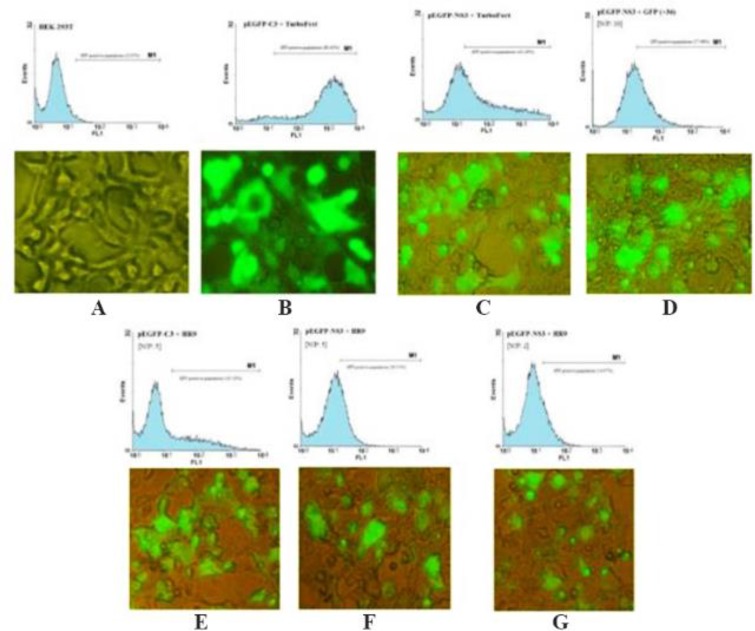
HR9 CPP-mediated NS3 DNA delivery in HEK-293T cells by Fluorescent microscopy and Flow cytometry: NS3 DNA was incubated with HR9 for 1 *hr*, with +36 GFP for 4 *hr* and TurboFect for 6 *hr*, respectively. A) HEK-293T as a negative control; B) pEGFP-C3/TurboFect as a positive control; C) pEGFP-NS3/TurboFect; D) pEGFP-NS3/+36 GFP at an N/P ratio of 10:1; E) pEGFP-C3/HR9 at an N/P ratio of 5:1; F) pEGFP-NS3/HR9 at an N/P ratio of 5:1; G) pEGFP-NS3/HR9 at an N/P ratio of 2:1.

**Figure 6.**
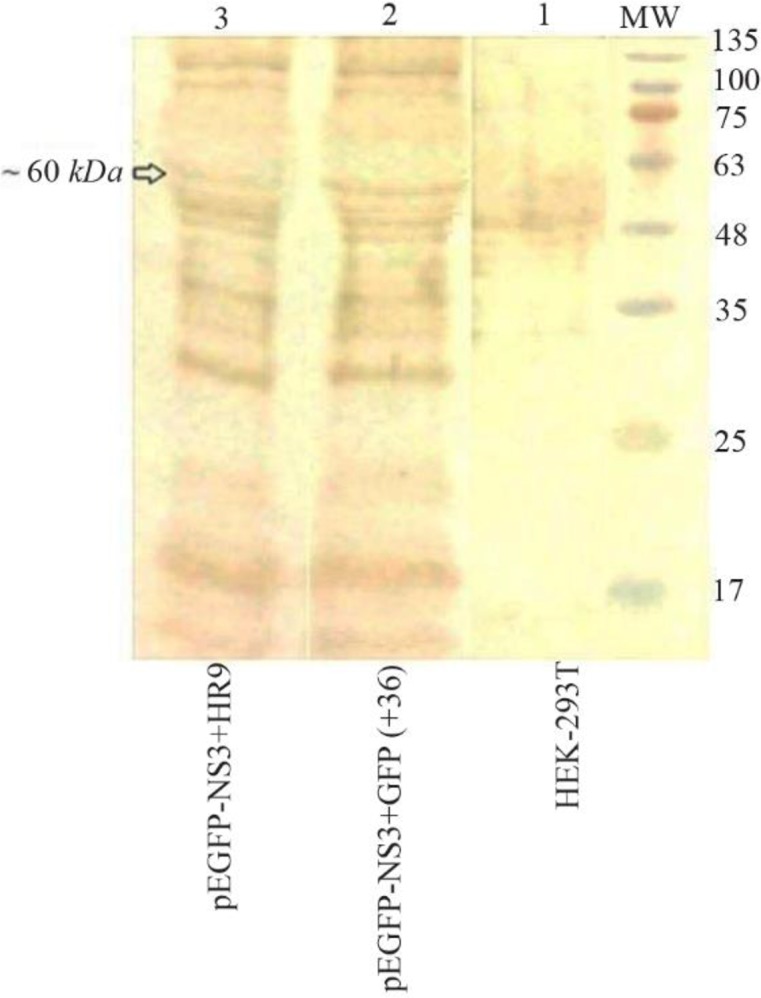
Western blot analysis of the fused NS3-GFP expressed in HEK-293T cells at 48 *hr* after transfection using an anti-GFP polyclonal antibody (Abcam); Lane 1: un-transfected cells, Lane 2: transfected cells with +36 GFP/NS3 DNA at N/P ratio of 10:1, Lane 3: transfected cells with HR9/NS3DNA complex at N/P ratio of 5:1. MW is the molecular weight marker (10–180 *kDa*, Fermentase). The dominant band of ∼60 *kDa* was detected in transfected cells with these complexes.

The ability of HR9 complexed with pEGFP-NS3 (at two N/P ratios: 2 and 5) was evaluated for penetration into HEK-293T mammalian cells. The expression of NS3 protein was confirmed by flow cytometry and fluorescence microscopy using GFP reporter marker in HEK-293T cells (Figure 5) 48 *hr* post-transfection. These results indicated that both +36 GFP and HR9 were able to deliver plasmid DNA into mammalian cells indicating HCV NS3 gene expression. The delivery of pEGFP-NS3 was detected in approximately 42.26±1.19 and 57.96±2.24 of HEK-293T cells treated with TurboFect and +36 GFP, respectively. In contrast, the pEGFP-NS3 delivery was detected in approximately 20.51±0.94 and 14.96±1.14 of HEK-293T cells treated with the HR9/NS3 DNA nanoparticles at N/P ratios of 5:1 and 2:1, respectively. The data indicated that the transfection efficiency of +36 GFP-based nanoparticles and TurboFect/NS3 DNA complexes was significantly higher than HR9/DNA in human cell line (p<0.05). The percentage of GFP-positive cells was 80.63±2.76 and 23.10±1.99 after transfection with pEGFP-C3+ TurboFect (as a positive control) and pEGFP-C3+HR9 complexes in two independent experiments, respectively. Thus, the percentage of NS3-GFP expression was not significantly different for HR9/NS3 DNA nanoparticles and HR9/pEGFP-C3 at N/P ratio of 5:1 in two independent experiments (p>0.05). The results indicated that arginine-rich CPP, HR9 was able to deliver the plasmid NS3 DNA non-covalently into HEK-293T cells. Fluorescent microscopy showed no detectable signal in the cells treated with the plasmid DNA alone (data not shown). In contrast, green fluorescence was observed in the cells treated with HR9/NS3 DNA and NS3DNA/+36 GFP complexes (Figure 5). These results indicated that CPP/ NS3 DNA nanoparticles had the ability to enter cells. The efficiency of the complexes was in the order of +36GFP/NS3 DNA>TurboFect/NS3 DNA>HR9/NS3 DNA complexes (Figure 5). Regarding previous results, zeta potentials of HR9/ NS3 DNA complexes were more electropositive than that of NS3 DNA as a more effective agent than their size. Thus, a more electropositive charge of HR9/NS3 DNA complexes led to the effective transfection of plasmid DNA. On the other hand, the efficiency of NS3 DNA delivery was investigated in the HEK-293T cells transfected by nanoparticles using western blotting at 48 *hr* post-transfection. The dominant band of ∼60 *kDa* was detected in transfected cells with these complexes using the anti-GFP polyclonal antibody (Figure 6). The corresponding bands were not detected in the un-transfected cells indicating that HR9 and +36 GFP could transfer NS3 DNA into the cells. However, the delivery of NS3 DNA by HR9 peptide was considerably lower than it by +36 GFP, thus the NS3 expression was detected for HR9/pEGFP-NS3 nanoparticles weaker than +36 GFP/ pEGFP-NS3 nanoparticles in western blotting.

## Discussion

Several controversial reports indicated the immunosuppressive effects of full NS3 protein on Antigen Presenting Cells (APCs) 18,19. It seems that the new vaccine design based on removing the enzymatic activity of NS3 and/or even the selection of truncated immunodominant fragments would be able to boost immune responses in APCs 20,21. In this study, a recombinant vector harboring the truncated and conserved NS3 gene was constructed. A study showed that zeta-potential of CPP/cargo complexes plays a major role in determining transduction efficiency, while their particle size has a minor effect on cell penetration. Moreover, the effects of zeta-potential and size of CPP/cargo complexes could lead to the stability of particles in solution 22. Our study also indicated the importance of zeta-potential of HR9/NS3 DNA nanoparticles compared to its size as assessed by Zetasizer. In a study, the pBacCecBE-GFP plasmid was mixed with HR9 at N/P ratios of 0, 0.2, 0.4, 0.6, 0.8, 1, 1.2 and 1.4. The DNA did not completely migrate at N/P:1.2 23. Our study also indicated that the plasmid harboring GFP gene alone (pEGFP-C3) complexed with HR9 did not migrate at N/P: 1; this ratio was increased for the plasmid harboring NS3-GFP fusion gene (pEGFP-NS3) complexed with HR9 (N/P: 2). Another study showed that the core of a red-emitting Quantum Dots (QD) was approximately 5.6 *nm* in diameter. Addition of HR9 to QD increased the size of HR9/QD complexes slightly ∼6.5 *nm*, while addition of SR9 and PR9 doubled the sizes of SR9/QD and PR9/ QD complexes. Indeed, HR9/QD complexes were more compact in structure than SR9/ QD and PR9/QD complexes. On the other hand, QDs indicated negative charges (−17.3 *mV*), while HR9/QD complexes displayed positive charges (+29.1 *mV*) indicating the importance of electropositive charges of CPP/QD complexes in cell transfection 24. Our data showed similar results. Furthermore, the addition of CPPs (SR9, HR9, and PR9) could similarly increase the size of green-emitting QDs (∼2.0 *nm*) to ∼15.7 *nm* in diameter 25. In this study, the N/P ratios of 2 and 5 were used as the combination ratio between HR9 CPP and plasmid DNA (pEGFP-NS3) in subsequent experiments. These results indicated that the HR9 CPP and HR9 CPP/NS3 DNA complexes showed no cytotoxicity in HEK-293T cells. Other studies also showed that CPPs alone or with their cargoes had no cytotoxicity in plant cells by Trypan blue or MTT assay. Generally, CPPs were non-toxic and non-immunogenic in human cell lines and in mice. These findings indicated that CPPs possess promising potential as safe and efficient nanocarriers (*i.e.*, without risks of toxicity and inflammatory reactions) in the delivery of cargo molecules into live cells 9. Moreover, it was observed that HR9 and HR9/QDs were not cytotoxic 26. It has also been shown that HR9 peptide and HR9/pEGFP-NS3 were not cytotoxic.

As known, due to the low penetration of plasmid DNAs into the cells, development of an effective adjuvant and/ or delivery system is necessary for designing DNA vaccines. For example, the studies indicated that intradermal delivery of DNA encoding HCV NS3 and a cytolytic protein (perforin) elicits robust cell-mediated immunity in mice and pigs 27. To facilitate gene transfer into cultured cells and living animals, a number of peptide carriers that combine DNA binding, such as electrostatic domain (polylysine and polyarginine), and membrane-destabilizing properties have been developed 10. A synthetic nona-arginine (SR9) CPP could deliver non-covalently DNAs into plant and human cells 28–30. Recently, two improved arginine-rich CPPs, histidine-rich nona-arginine (HR9) and Pas nona-arginine (PR9) could deliver DNAs into paramecia 31 or insect cells 23, or deliver nanoparticles 32,33 and proteins 34 into human cells. Herein, HEK-293T cells could express HCV *NS3* gene after transfection with HR9 peptide. The transfection rate for NS3DNA/HR9 nanoparticles was significantly lower than NS3DNA/ +36GFP nanoparticles and NS3DNA/TurboFect complexes (p<0.05). However, it is important that HR9 could deliver NS3 DNA in lower ratio and incubation time than +36 GFP. The lower size of HR9/NS3 DNA (100–150 *nm*) could lead to the direct and quick penetration of NS3 DNA into the cells compared to +36 GFP/NS3 DNA (200–250 *nm*). In a study, arginine-rich CPPs (PR9, SR9 and HR9) were used to deliver genetic material into target cells. The data indicated that HR9 was superior, likely due to the histidine facilitated DNA endosomal escape 35. Another report showed that histidine-modified arginine-rich CPP, HR9 could facilitate the cellular uptake of the fluorescent nanodiamonds. In fact, a combined use of CPPs and nano-scaled materials (with or without fluorescence) could significantly enhance efficiency for imaging and therapeutic uses 36. In general, delivery of semiconductor QDs conjugated with CPPs into cells by the endocytic pathway was problematic in biomedical applications because of lysosomal trapping. HR9 peptides stably and non-covalently combined with QDs were able to enter into cells in a short time (4 *min*) indicating that HR9 penetrated cell membrane directly 26. In the present study, HR9 peptide was able to form stable complexes with NS3 DNA, and the complexes could effectively internalize into human cells. This delivery time is less than +36 GFP and was performed at lower ratios of HR9/cargo (N/P: 2 or 5) compared to +36 GFP/cargo (N/P=10:1). A study also demonstrated that three arginine-rich CPPs (SR9, HR9, and PR9) were able to transport plasmid DNA into human A549 cells. Mechanistic studies revealed that HR9/DNA complexes mediate the direct membrane translocation pathway for gene delivery 35. On the other hand, two arginine-rich CPPs, HR9 and IR9, were able to efficiently and non-covalently deliver plasmid DNAs, Red Fluorescent Proteins (RFPs), and semiconductor QD into rotifers. Indeed, HR9-delivered plasmid DNAs containing the enhanced GFP and RFP coding sequences could be actively expressed in rotifers 37. Moreover, GFP either alone or non-covalently associated with a CPP comprised of nine arginine residues (R9/GFP complexes) entered cyanobacteria. This CPP-mediated delivery system was not toxic to cyanobacteria, and could be used to investigate biological processes at the cellular level in this species^38^.

Other studies also demonstrated that arginine-rich CPPs (SR9, HR9 and PR9) were able to deliver the plasmid DNA into Sf9 cells, and the delivered DNA could be expressed subsequently. It was found that the efficiency of gene delivery differed among CPPs with the order of HR9 (6.67±0.94%) >PR9 (4.00±0.82%) >SR9 (3.33±0.47%) at N/P ratio of 3:1. Moreover, the percentage of transfected cells was calculated as HR9 (6.16±0.89%) >PR9(3.90±0.71%) >SR9(0.19±0.09%) at N/P ratio of 5:1 23,39. Indeed, the efficiency of transfection was not different at two N/P ratios as observed in our study. Altogether, our study showed that the HR9-delivered plasmid encoding the NS3-GFP fusion gene could be successfully expressed by HEK-293T cells.

## Conclusion

In summary, HR9 interacted with NS3 DNA to form stably non-covalent complexes in vitro. This CPP enhanced the delivery of associated DNA into human HEK-293T cells. Zeta-potential and size analyses indicated the importance of electrostatic interactions between HR9/DNA nanoparticles and cytoplasmic membranes. Furthermore, the HR9 peptide and HR9/DNA complexes were not cytotoxic at certain concentrations. Our results suggested that HR9 can be considered a highly efficient and promising tool for gene transfer in HCV vaccine development. However, it is required to evaluate *in vivo* gene delivery using HR9 peptide in near future.
